# Eye Protection Chamber for Preventing Corneal Injury in Surgical Patients: Protocol for a Randomised Pilot and Feasibility Trial

**DOI:** 10.1002/nop2.70964

**Published:** 2026-10-22

**Authors:** Thalita Gomes do Carmo, Roberto de Souza, Anna Beatriz Justen dos Santos, Luana Faria Barbosa, Giovanna Oliveira de Marteloti, Maria Eduarda Maciel Palmares, Eduardo Melani Rocha, Patricia Rezende do Prado

**Affiliations:** ^1^ Department of Medical‐Surgical Nursing Universidade Federal Fluminense (Fluminense Federal University) Niterói Rio de Janeiro Brazil; ^2^ Instituto Nacional de Cardiologia (National Institute of Cardiology) Rio de Janeiro Rio de Janeiro Brazil; ^3^ School of Nursing Universidade Federal Fluminense (Fluminense Federal University) Niterói Rio de Janeiro Brazil; ^4^ Resident in Obstetric Nursing Universidade do Estado do Rio de Janeiro (Rio de Janeiro State University) Rio de Janeiro Rio de Janeiro Brazil; ^5^ Department of Ophthalmology, Otorhinolaryngology, and Head and Neck Surgery Ribeirão Preto Medical School, University of São Paulo Ribeirão Preto São Paulo Brazil; ^6^ Department of General and Specialized Nursing Ribeirão Preto College of Nursing, University of São Paulo Ribeirão Preto São Paulo Brazil

**Keywords:** corneal abrasion, eye protection chamber, general anaesthesia, patient safety, perioperative nursing, randomised controlled trial

## Abstract

**Aim:**

To assess the feasibility of conducting a definitive randomised trial comparing an eye protection chamber with standard microporous adhesive tape in adults undergoing general anaesthesia for surgery expected to last more than 120 min, and to obtain preliminary estimates of perioperative corneal‐injury event rates and device‐related outcomes.

**Methods:**

This single‐centre, parallel‐group, assessor‐blinded, randomised pilot and feasibility trial will allocate participants using simple randomisation with a 1:1 allocation probability to either the control group (adhesive tape and lubricating eye drops) or the intervention group (lubricating eye drops and an eye protection chamber). Primary feasibility outcomes will include recruitment, retention and completeness of the planned corneal assessments, intervention adherence, device displacement/reapplication and safety. Corneal examinations using fluorescein staining and slit‐lamp assessment will be performed before induction of general anaesthesia (T0), during the immediate postoperative period after completion of surgery and removal of the eye‐protection device (T1), and 24 h after surgery (T2). The incidence of any new corneal epithelial injury detected at T1 and/or T2 that was absent at T0 will be estimated as an exploratory clinical outcome. A pragmatic total sample of 120 participants will be retained to estimate feasibility parameters and preliminary event rates; the trial is not powered for confirmatory superiority testing.

**Patient or Public Contribution:**

No patient or public involvement occurred in the development of this protocol. Nursing relevance: The trial is intended to inform the design of a definitive study and may support the development of evidence‐based perioperative eye‐protection protocols.

**Trial Registration:**

Brazilian Registry of Clinical Trials (Registro Brasileiro de Ensaios Clínicos [ReBEC]) under UTN number U1111‐1285‐5334

## 1 Introduction

General anaesthesia suppresses physiological mechanisms that protect the ocular surface, including blinking, complete eyelid closure, Bell's phenomenon and tear production. Disruption of the precorneal tear film can expose the corneal epithelium to desiccation and mechanical trauma during surgery (Stapleton et al. 2025). Corneal abrasion is the most frequently reported perioperative ocular injury and may cause pain, photophobia, blurred vision, delayed recovery and rarely, persistent visual morbidity (Moos and Lind 2006; Papp et al. 2019).

Reported incidence varies markedly across studies. A recent systematic review identified corneal injury rates ranging from 0.01% to 59% in surgical patients, depending on the population studied, the eye protection strategies employed and, notably, the diagnostic method used (Papp et al. 2019).

Wan et al. (2014) reported a corneal injury incidence of 39.5% in surgical patients treated with adhesive tape, compared with 15.8% in those treated with a hydrogel eye dressing. Although the hydrogel dressing differs from the polyethylene eye protection chamber evaluated in the present study, these findings illustrate the variability in event rates associated with different ocular‐protection strategies and should not be used as a confirmatory effect estimate for the chamber.

Standard perioperative eye care usually combines lubricating ophthalmic drops with eyelid closure using adhesive tape. Tape is inexpensive and familiar, but it may loosen during repositioning, fail to maintain complete closure in participants with lagophthalmos, irritate periorbital skin or require reapplication, thereby increasing nursing workload and interrupting workflow (Papp et al. 2019; Liyew et al. 2024). A moisture‐retaining eye protection chamber may reduce evaporation and mechanical exposure while maintaining eyelid closure.

A pilot randomised controlled trial in 80 critically ill adults randomised 40 participants to eye drops plus a polyethylene chamber and 40 to eye drops plus adhesive tape. Corneal injury was reported in 6 participants in the tape group and 3 participants in the chamber group (Prado et al. 2026). Because this was a pilot study conducted in critically ill patients, these event counts are not treated as a definitive effect estimate for the present surgical population. Critically ill patients differ from elective surgical patients in duration of sedation, organ dysfunction, mechanical ventilation and repeated eye‐care exposure. These data therefore support the need for perioperative feasibility and event‐rate estimates before planning a definitive confirmatory trial.

Perioperative nurses are responsible for applying eye‐protection measures, monitoring their integrity and identifying ocular adverse events. A randomised pilot and feasibility trial can establish whether recruitment, intervention delivery, outcome ascertainment and safety monitoring are workable in the perioperative setting, while generating preliminary event‐rate estimates needed to design a future definitive trial.

## 2 Objectives

### 2.1 Primary Objective

To assess the feasibility of conducting a definitive randomised trial comparing an eye protection chamber with adhesive tape for perioperative ocular protection in adults undergoing general anaesthesia for surgery expected to last more than 120 min.

### 2.2 Secondary Objectives

- To estimate recruitment, retention and completeness of the planned corneal assessments.
- To characterise intervention adherence, device displacement/reapplication and prespecified device‐related adverse events.
- To estimate the incidence, severity and laterality of new perioperative corneal epithelial injury in each group and to obtain exploratory between‐group effect estimates; participant‐ and procedure‐related risk‐factor analyses will be hypothesis‐generating.

### 2.3 Exploratory Clinical Comparison

Between‐group differences in corneal injury will be treated as exploratory estimates to inform a future definitive trial. No confirmatory superiority hypothesis will be tested in this pilot and feasibility study.

## 3 Methods

### 3.1 Protocol Reporting and Trial Design

This protocol follows the SPIRIT 2025 Statement and its Explanation and Elaboration document (Chan et al. 2025; Hróbjartsson et al. 2025). It describes a single‐centre, two‐arm, parallel‐group, assessor‐blinded, randomised pilot and feasibility trial with individual participant allocation in a 1:1 ratio.

No patient or public involvement occurred in the development of this protocol.

No confirmatory economic evaluation is planned. Device displacement, reapplication and related nursing actions will be recorded as feasibility/process measures; any formal collection of nursing time or cost data must be specified before recruitment begins.

### 3.2 Trial Setting

The trial will be conducted in the operating rooms and immediate postoperative care areas of Hospital Universitário Antônio Pedro, a public hospital within the Brazilian Unified Health System (Sistema Único de Saúde [SUS]) and managed by the Brazilian Hospital Services Company (Empresa Brasileira de Serviços Hospitalares [Ebserh]), located in the municipality of Niterói, Rio de Janeiro State, Brazil.

Population: Participants undergoing general anaesthesia for surgical procedures longer than 120 min.

### 3.3 Participants

#### 3.3.1 Inclusion Criteria

- Adults aged 18 years or older.
- Scheduled for non‐ocular surgery under general anaesthesia.
- Expected surgical duration longer than 120 min.
- Able to provide written informed consent, or represented by a legally authorised representative when permitted by the approved protocol.

#### 3.3.2 Exclusion Criteria

- Corneal epithelial injury detected during the preoperative fluorescein examination.
- Emergency polytrauma surgery when consent cannot be obtained.
- Impaired consciousness (stupor or coma) without an available legally authorised representative able to provide consent.

### 3.4 Recruitment, Withdrawal and Retention

Potential participants will be identified from the surgical schedule and screened before the procedure. The research team will document the number assessed, reasons for exclusion, number eligible, number consenting, number randomised, and reasons for non‐retention. Recruitment rate and retention/completion of the planned outcome assessments will be summarised as feasibility outcomes. Recruitment is planned over 12 months, with a target average recruitment rate of 10 randomised participants per month. Participants may withdraw consent at any time without affecting their care.

A participant will be considered not to have completed the planned follow‐up if the operation is cancelled or aborted after randomisation and the postoperative corneal assessments cannot be completed, the participant declines a postoperative examination, or either the T1 or T2 assessment cannot otherwise be obtained. Reasons will be recorded. All randomised participants will remain documented according to their original randomised group; available outcome data will be retained unless consent for data use is withdrawn.

Based on institutional surgical activity, approximately 400 patients per year undergo general anaesthesia for procedures expected to last longer than 120 min at the study site.

### 3.5 Sample Size

This study is designed as a pilot and feasibility trial rather than a confirmatory superiority trial; therefore, no formal hypothesis‐testing sample‐size calculation is used to establish efficacy. The previous calculation based on Wan et al. (2014) was withdrawn because that study evaluated a hydrogel eye dressing rather than the polyethylene eye protection chamber and assumed substantially higher event rates than those reported in the authors' previous chamber pilot. A pragmatic target of 120 participants will be retained, with a 1:1 allocation probability under simple randomisation, to estimate recruitment, retention, intervention adherence, assessment completeness, safety and preliminary corneal‐injury event rates and effect estimates. Because simple randomisation does not force equal final group sizes, the realised numbers in the two groups may differ from 60/60. These estimates will be used to inform the design and sample‐size calculation of a future definitive trial.

The planned sample size of 120 participants should not be interpreted as providing adequate statistical power to demonstrate or exclude superiority of the eye protection chamber.

### 3.6 Randomisation and Allocation Concealment

Before participant enrolment begins, the trial statistician, who will not be involved in participant screening, consent, intervention delivery, outcome assessment or the blinded clinical analysis, will generate a computer‐based simple randomisation sequence for 120 allocation positions. Each allocation will be generated independently with a probability of 0.50 for control and 0.50 for intervention. No forced balance, block randomisation or stratification will be used. The trial statistician will retain the randomisation file and generation details as trial documentation and will prepare the sequentially numbered, opaque, sealed envelopes according to the generated sequence. The trial statistician will take no part in participant enrolment or outcome assessment.

Sequentially numbered, opaque, sealed envelopes will be opened in numerical order only after eligibility has been confirmed, written informed consent has been obtained and the baseline corneal examination has been completed. The assistant researcher responsible for allocation will open the next numbered envelope and will not perform participant enrolment or ocular outcome assessment.

Upcoming allocations and the randomisation list will remain concealed from research‐team members involved in enrolment, intervention delivery and outcome assessment until assignment. Any skipped, damaged or prematurely opened envelope will be documented as a protocol deviation, preserving an auditable allocation trail.

### 3.7 Blinding

Participants will be under general anaesthesia when the assigned device is applied and will not be informed of allocation before the postoperative corneal assessments. Nurses applying the chamber or tape cannot be blinded because the devices are visibly different. Application, displacement, reapplication and protocol deviations will be documented. The trial statistician who generates the randomisation sequence will not conduct the clinical analyses. A second statistician, who will have no access to the randomisation sequence or allocation key, will conduct the prespecified analyses using masked group labels (Group A and Group B). The allocation code key will be retained by the assistant researcher responsible for allocation and will not be released to the analysing statistician until the prespecified analyses have been completed.

Before the postoperative T1 assessment, a nurse who is not the outcome assessor will remove the eye‐protection device and perform periorbital cleansing before the assessor enters the assessment area. Packaging, devices and intervention records will be kept out of the assessor's view. The outcome assessor will not be present during device removal or periorbital cleansing, thereby reducing the possibility of identifying group allocation.

### 3.8 Interventions

Both groups will receive identical baseline ocular lubrication with 0.5% sodium carmellose ophthalmic solution. In the control group, eyelid closure will be achieved using adhesive tape, whereas in the intervention group, ocular protection will be provided using the eye protection chamber (EPC). Thus, the only difference between the groups will be the eyelid occlusion method.

#### 3.8.1 Control Group: Adhesive Tape

After induction of general anaesthesia and loss of the blink reflex, the perioperative nurse will instil the lubricating drops and close both eyelids completely with microporous adhesive tape. The tape will remain in place during surgery unless displacement, contamination, incomplete closure or a safety concern requires reapplication. It will be removed after surgery before the blinded corneal assessment.

#### 3.8.2 Intervention Group: Eye Protection Chamber

After induction of general anaesthesia and loss of the blink reflex, the perioperative nurse will instil the identical lubricating drops and apply the flexible polyethylene eye protection chamber over the closed eyelids. The chamber will remain in place during surgery unless displacement, loss of seal, contamination or a safety concern requires reapplication. It will be removed after surgery before the blinded corneal assessment.

### 3.9 Periorbital Cleansing and Adherence Monitoring

The study does not prescribe a formally standardised periocular cleansing protocol specifying gauze dimensions, number of wipes, or a fixed cleansing sequence. Periorbital cleansing will be performed after device removal and before the outcome assessor enters the assessment area. Application‐related adherence events, including incomplete eyelid closure, displacement, slippage, reapplication and unplanned removal, will be documented during the intraoperative period.

Consent for publication of identifiable images: The individual depicted in Figures 1, 2, 3 is the principal investigator of this study and has consented to publication of these images.

**FIGURE 1:**
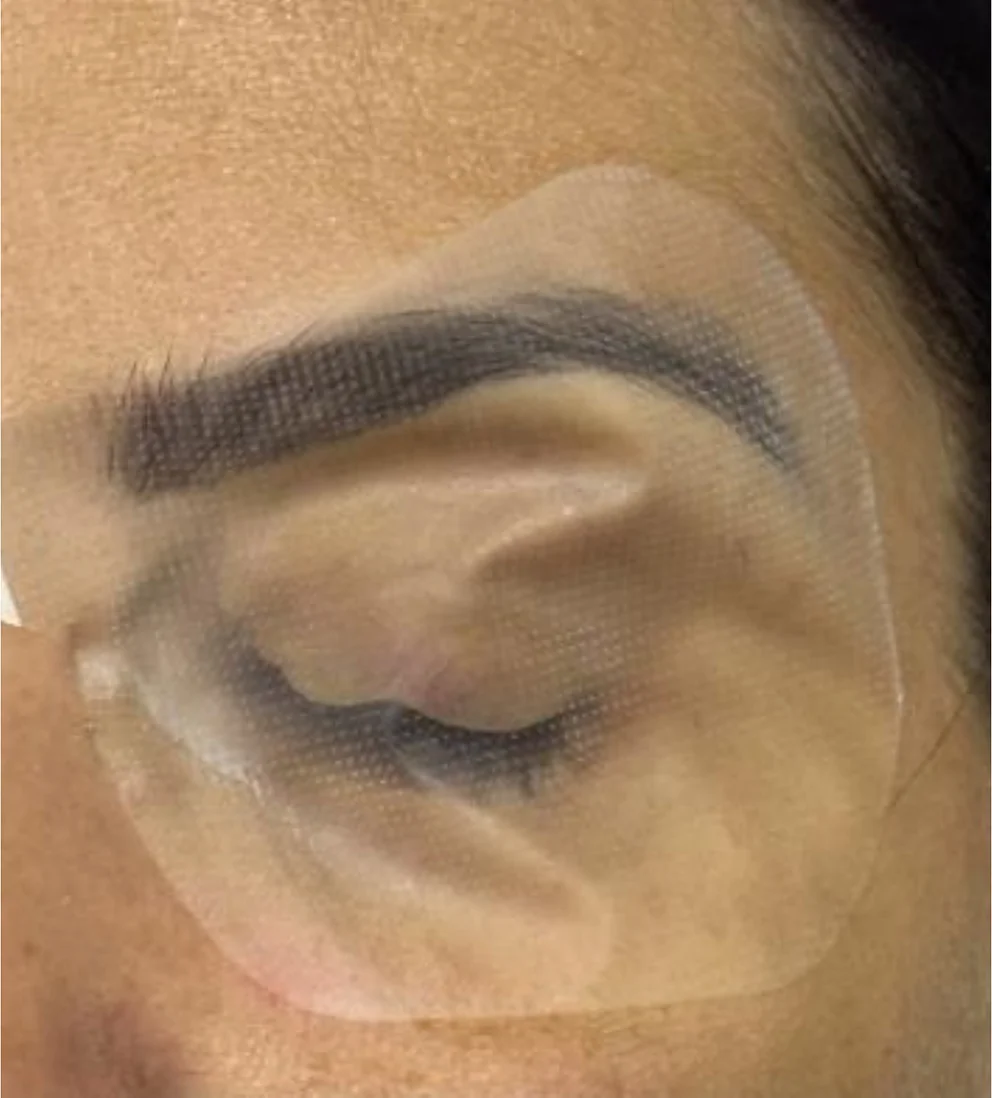
Polyethylene eye protection chamber for ocular occlusion.

**FIGURE 2:**
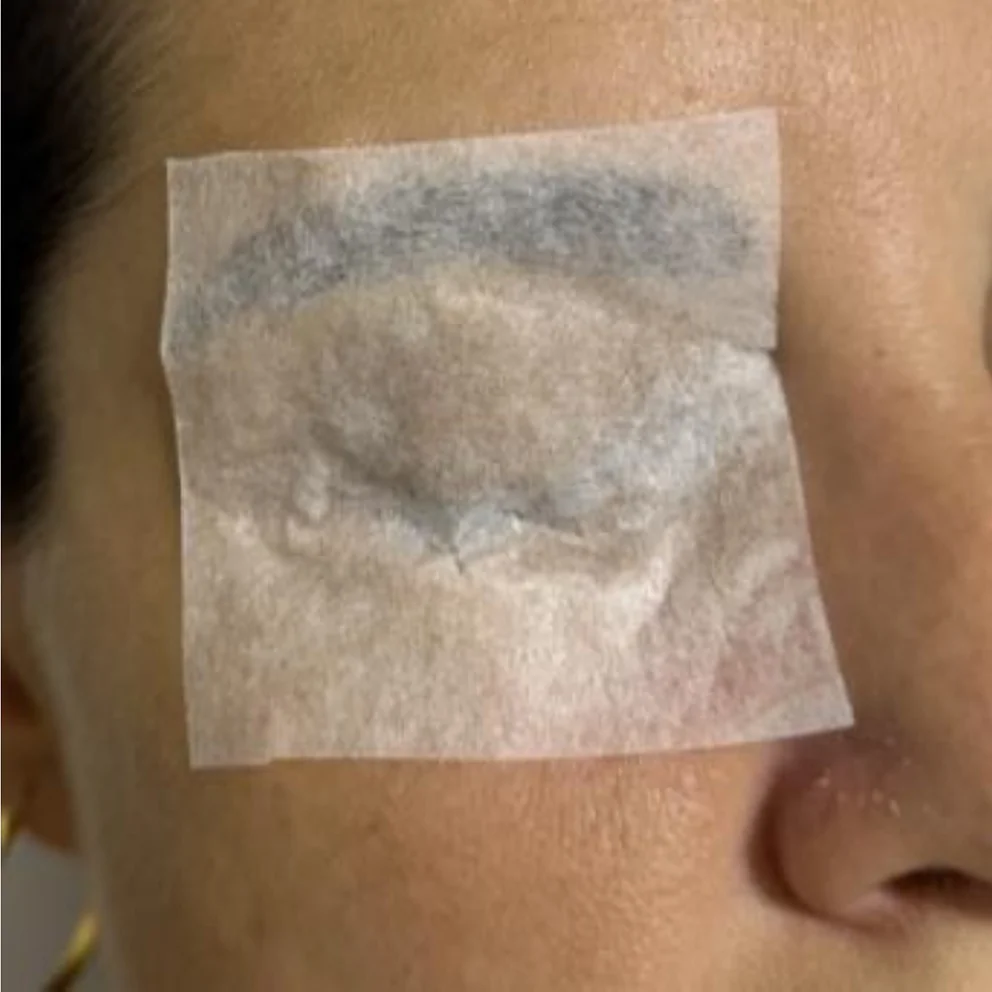
Adhesive tape used for ocular occlusion.

**FIGURE 3:**
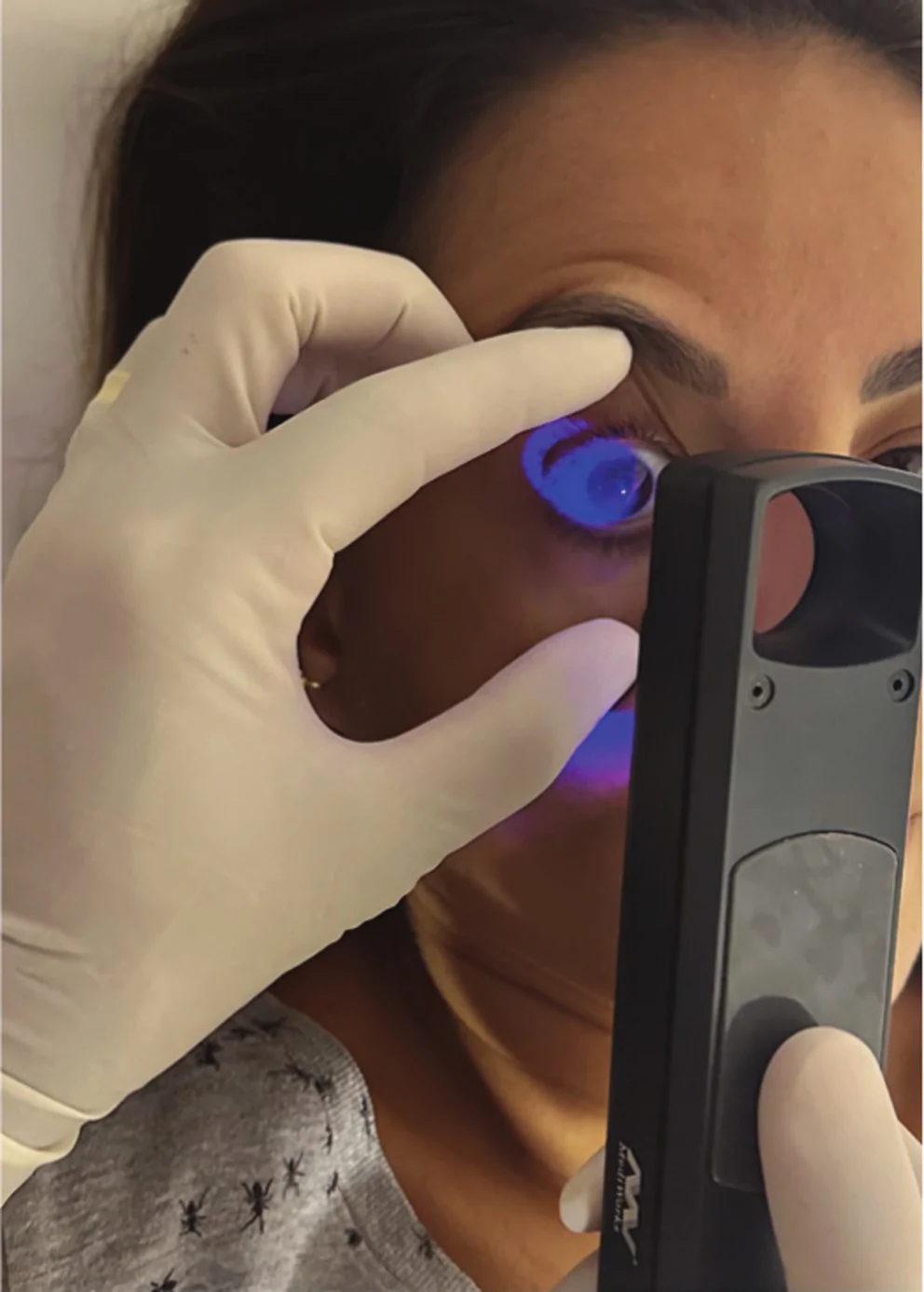
Corneal examination using a slit lamp.

### 3.10 Feasibility Outcomes and Clinical Outcome Assessment

Primary feasibility outcomes will include recruitment rate; retention and completeness of the planned T1 and T2 corneal assessments; intervention adherence; device displacement, slippage, reapplication or unplanned removal; and the occurrence of prespecified adverse events. These outcomes will be summarised by group and judged against prespecified progression criteria to determine whether a future definitive trial should proceed unchanged, proceed with protocol modification, or be reconsidered.

#### 3.10.1 Progression Criteria

Progression to a future definitive randomised trial will be assessed after the 12‐month recruitment period using a traffic‐light framework. Green indicates that the definitive trial may proceed using the current procedures; amber indicates that progression may be appropriate after targeted protocol modification; and red indicates that progression should not occur without major redesign. Overall progression will be classified as green only if all domains are green and no safety red criterion is met; amber if one or more domains are amber and none is red; and red if any red criterion is met. Recruitment will be judged across the whole trial. Retention, assessment completeness, intervention adherence and device displacement/reapplication will be calculated separately for each randomised arm, and the less favourable arm‐specific category will determine the domain classification. Safety events will be summarised by arm and across the whole trial; any safety red criterion in either arm will classify the safety domain as red. Threshold classification will use the exact unrounded numerator/denominator proportion; displayed percentages will be rounded to one decimal place.

**Table:** 

Feasibility domain	Green—proceed	Amber—amend	Red—reconsider/stop
Recruitment over 12 months (overall)	120 participants recruited (average ≥ 10/month)	96–119 participants recruited (average 8.0–9.9/month)	<96 participants recruited (average < 8/month)
Retention through T2 (per arm; worse arm determines category)	≥ 90%	80%–89%	< 80%
Complete T1 and T2 corneal assessments (per arm; worse arm determines category)	≥ 90%	80%–89%	< 80%
Intervention adherence (per arm; worse arm determines category)	≥ 90%	80%–89%	< 80%
Device displacement/reapplication (per arm; worse arm determines category)	≤ 10%	> 10%–20%	> 20%
Safety (by arm and overall)	No serious intervention‐related adverse event	Non‐serious intervention‐related events indicate need for protocol modification	Any serious intervention‐related adverse event or safety signal requiring study suspension

Operational definitions for progression criteria: recruitment is the number randomised during the 12‐month recruitment period relative to the target of 120 participants. Retention through T2 is the number of randomised participants with follow‐up status established at T2 divided by the number randomised in that arm. Complete T1 and T2 corneal assessment is the number of randomised participants with evaluable fluorescein/slit‐lamp examinations at both T1 and T2 divided by the number randomised in that arm. An adherent participant is one who receives the allocated ocular‐protection method after induction, with complete eyelid closure achieved, without crossover to the alternative study method or unplanned permanent discontinuation; protocol‐permitted reapplication of the same assigned method after displacement does not constitute non‐adherence. The adherence denominator is the number of participants in that arm whose surgery proceeds to application of the assigned intervention. Device displacement/reapplication is the number of participants with at least one displacement, slippage, reapplication or unplanned removal divided by the number receiving the assigned intervention in that arm. A serious intervention‐related adverse event is an event judged by the principal investigator to be at least possibly related to the chamber, adhesive tape or a study procedure and that results in death, is life‐threatening, requires or prolongs hospitalisation, causes persistent or significant disability/incapacity, or is otherwise medically important and requires intervention to prevent one of these outcomes. A corneal ulcer will always be recorded as an exploratory clinical outcome and as an adverse event; it will be classified as a serious intervention‐related adverse event only when it meets the seriousness criteria and is judged at least possibly related to the study intervention or procedure.

The exploratory clinical outcome is the occurrence of any new corneal epithelial injury in either eye detected at the immediate postoperative assessment (T1) and/or at the 24‐h postoperative assessment (T2) that was absent at the preoperative baseline examination (T0). Injury will be assessed by fluorescein staining under cobalt‐blue illumination using a slit lamp. Corneal epithelial lesions will be classified according to morphological presentation as: (1) punctate epithelial erosions, characterised by fine, scattered fluorescein‐positive spots; (2) corneal abrasion or macro‐epithelial defect, characterised by a larger, confluent area of epithelial loss; or (3) corneal ulceration (Sanadgol et al. 2025). Any newly detected lesion not present at baseline will constitute a clinical event. Lesion type, laterality, dimensions and the assessment time at which the lesion is first detected will be recorded. No predefined dimensional threshold will be used to distinguish categories.

The participant is the unit of analysis for the clinical event. A participant with injury in one or both eyes will contribute one participant‐level event. Laterality will be described as unilateral or bilateral; bilateral injuries will not be counted as two independent participant‐level events.

The data‐collection instrument has reported face validity in earlier work. Separate evidence using fluorescein assessment by trained nurses found almost perfect inter‐rater agreement, with kappa values from 0.84 to 0.93 (Araujo et al. 2017). This supports reliability after training but does not establish diagnostic sensitivity or specificity against an ophthalmologist.

The primary outcome assessor will be a perioperative nurse specialist with more than 10 years of operating room experience and doctoral‐level training. The assessor completed one month of specific training with an ophthalmologist in corneal assessment and identification of suspected corneal injury using fluorescein staining and slit‐lamp examination. The assessor will be independent of intervention delivery and blinded to treatment allocation.

### 3.11 Assessment Schedule and Follow‐Up

Corneal assessment will be performed at three predefined time points: T0, during the preoperative period before induction of general anaesthesia; T1, during the immediate postoperative period after completion of surgery and removal of the eye protection device; and T2, 24 h after surgery. The T1 assessment will be performed after periorbital cleansing and before the outcome assessor is informed of any information that could reveal treatment allocation. The T2 assessment will use the same fluorescein staining and slit‐lamp examination procedure.

The exploratory clinical endpoint will be any new corneal epithelial injury detected at T1 and/or T2 that was absent at T0. Inclusion of the 24‐h T2 assessment is intended to reduce underascertainment of epithelial injuries that may not be readily detectable during the immediate postoperative assessment.

### 3.12 Participant Timeline

The schedule of enrolment, allocation, interventions and assessments is presented in Table 1.

**TABLE 1:** Schedule of enrolment, allocation, interventions and assessments (SPIRIT 2025).

Procedure	Screening/preoperative (T0)	Allocation/before surgery	After induction/intraoperative period	Immediate postoperative period (T1)	24 h postoperative (T2)
Eligibility assessment and informed consent	X				
Baseline variables and corneal examination	X				
Randomisation		X			
0.5% sodium carmellose ophthalmic solution + assigned eye‐occlusion method			X		
Intervention adherence monitoring			X		
Adverse‐event monitoring			X	X	X
Device removal and periorbital cleansing				X	
Fluorescein/slit‐lamp corneal assessment	X			X	X
New corneal injury ascertainment				X	X
Final exploratory clinical‐event classification					X
Retention/follow‐up completion					X

### 3.13 Flowchart 1 Trial Pathway and Outcome Classification

**FLOWCHART 1:**
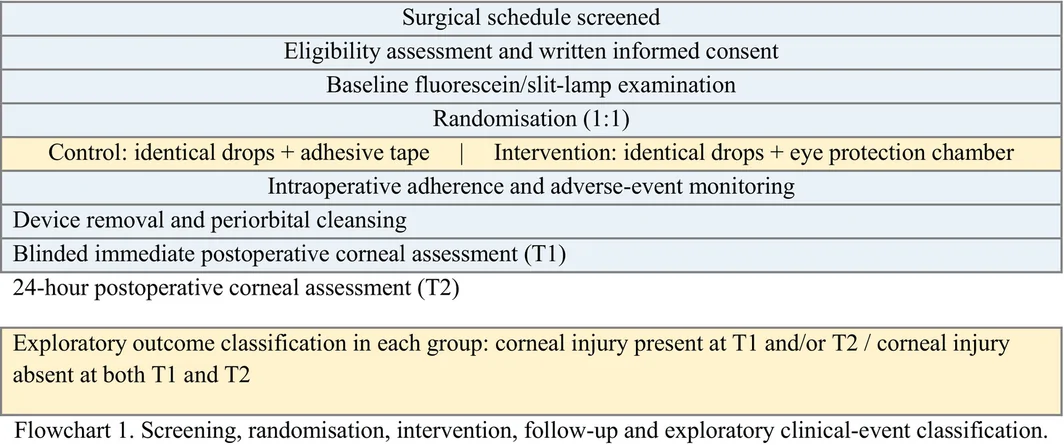
Screening, randomisation, intervention, follow‐up and exploratory clinical‐event classification.

### 3.14 Additional Outcomes and Covariates

Additional outcomes include lesion morphology and laterality, periorbital erythema, suspected adhesive allergy, eyelid indentation or compression injury, conjunctival irritation, device displacement and device reapplication. Adverse‐event incidence is not known reliably for this specific surgical comparison and will be estimated with 95% confidence intervals rather than assigned unsupported expected rates.

Analyses of participant‐ and procedure‐related factors associated with corneal injury will be exploratory. Candidate variables will be defined a priori before analysis on the basis of clinical relevance and previous evidence rather than by automated statistical selection. Surgical specialty will be described as part of the study population. The final case report form and operational definitions will be established before enrolment.

### 3.15 Harms and Safety Management

Adverse events will be monitored during the intraoperative period and at T1 and T2. Targeted surveillance will include periorbital erythema, skin reaction, eyelid indentation, conjunctival irritation, pain or visual symptoms, and any corneal lesion. Events will be documented with their timing, severity and potential relationship to the eye protection chamber, adhesive tape, fluorescein assessment or other study procedures and will be reviewed by the principal investigator. Any unexpected adverse reaction or safety concern potentially related to the chamber, adhesive tape, fluorescein ophthalmic solution or another study procedure will prompt suspension of the relevant study procedures while the event is clinically evaluated and reported to the Research Ethics Committee in accordance with applicable requirements.

### 3.16 Data Management and Confidentiality

Each participant will be assigned a unique numerical study code, which will be used in the research database instead of personal identifiers. Study data will be entered into REDCap, a password‐protected electronic data‐capture system with role‐based access and an electronic audit trail recording data creation and modification. Range and consistency checks will be incorporated into the database. Access will be restricted to authorised members of the research team, and the principal investigator will remain responsible for data custody. Research data will be retained for 5 years, in accordance with the recommendations of the Research Ethics Committee, and will subsequently be securely disposed of. Coded data will be used for statistical analysis and reporting, thereby preserving participant confidentiality throughout the study. Individual participant data will not be publicly shared or made available to third parties under the current data‐management plan.

### 3.17 Statistical Analysis

Continuous variables will be summarised using mean and standard deviation or median and interquartile range, as appropriate. Categorical variables will be presented as absolute and relative frequencies. Feasibility outcomes will be reported descriptively by group, including recruitment, retention, T1/T2 assessment completeness, adherence, device displacement/reapplication and adverse events.

Corneal‐injury event rates will be estimated for each group with exact Clopper‐Pearson 95% confidence intervals. A participant will be classified as having the exploratory clinical event if a new injury is detected at either T1 or T2. Exploratory between‐group effects will be reported as relative risk (RR) with a 95% Koopman asymptotic score confidence interval and absolute risk difference (ARD) with a 95% Newcombe hybrid‐score confidence interval based on Wilson score intervals. Fisher's exact test may be reported as an exploratory comparison, but no confirmatory superiority conclusion will be based on statistical significance. The clinical results will be interpreted primarily as estimates for planning a future definitive trial, and this pilot study will not be considered capable of excluding clinically important between‐group differences.

All randomised participants will constitute the intention‐to‐treat population and will remain documented according to their original allocation. Participants with a new corneal injury detected at T1 or T2 will be classified as having an event. Participants with no injury at T1 will require an evaluable T2 assessment to be classified as having no event; otherwise, the exploratory clinical outcome will be considered missing. Reasons for missing T1 or T2 data will be reported by group, and missing outcomes will not be imputed as injury or no injury. No separate per‐protocol efficacy analysis is planned; protocol deviations and intervention adherence will instead be summarised as feasibility outcomes.

Because few corneal‐injury events are anticipated, no multivariable adjustment model will be prespecified for the exploratory treatment‐effect analysis. Analyses of factors associated with corneal injury will be hypothesis‐generating only, with candidate variables defined a priori on the basis of clinical relevance and prior evidence rather than automated stepwise procedures or bivariate *p*‐value thresholds.

SPSS software, version 22.0, will be used for descriptive analyses and exploratory hypothesis testing. R statistical software (R Foundation for Statistical Computing, Vienna, Austria) will be used to calculate the prespecified Clopper‐Pearson, Koopman score and Newcombe hybrid‐score confidence intervals.

### 3.18 Trial Oversight and Monitoring

No independent Data Monitoring Committee (DMC) has been established. Trial conduct will be overseen by the principal investigator together with a multidisciplinary study‐monitoring group comprising members of the perioperative nursing team, the Center for Injury Prevention Research and Development (Cecipred), Supera Parque, the ophthalmology team and the statistics team. The principal investigator will have primary responsibility for participant safety and for the assessment, documentation and reporting of adverse events. No interim efficacy analysis is planned, and no fixed schedule of monitoring meetings has been established; safety and protocol adherence will be monitored throughout trial conduct.

If an unexpected adverse reaction or safety concern potentially related to the eye protection chamber, adhesive tape, fluorescein ophthalmic solution or another study procedure occurs, the relevant study procedures will be suspended while the event is clinically evaluated. The principal investigator will be responsible for documenting and managing the event and for reporting it to the Research Ethics Committee in accordance with applicable requirements. Continuation of the study will depend on participant‐safety assessment and any recommendations issued by the Research Ethics Committee.

## 4 Ethics and Dissemination

### 4.1 Ethical Approval and Informed Consent

The study will be conducted in accordance with the Declaration of Helsinki. Written informed consent will be obtained before any trial‐specific procedure by a trained member of the research team. Participants who can provide consent will provide written informed consent personally. For patients with impaired consciousness (stupor or coma) who are unable to provide informed consent, written informed consent may be obtained from a legally authorised representative in accordance with the approved protocol. Patients with impaired consciousness for whom no legally authorised representative is available will not be enrolled. Participants or their legally authorised representatives, as applicable, will receive information about the purpose, procedures, foreseeable risks, potential benefits, confidentiality, voluntary participation and the right to withdraw without affecting clinical care.

Trial registration: The registry name, registration identifier and registration date are provided on the separate Title Page and are omitted from this anonymised manuscript in accordance with the journal's blinded‐review requirements. The trial was registered before participant enrolment. No participant has been enrolled to date; recruitment will begin only after acceptance of this protocol.

### 4.2 Conflicts of Interests

The authors declare no conflicts of interests.

### 4.3 Ancillary Care and Compensation for Harm

Participants who develop ocular symptoms or an abnormal corneal examination will receive prompt clinical assessment and referral to ophthalmology according to institutional procedures. Immediate and comprehensive healthcare required for any research‐related injury will be provided without cost to the participant, in accordance with applicable Brazilian ethical and legal requirements. Participants retain the right to compensation for harm resulting from their participation in the research, as provided under applicable Brazilian regulations.

### 4.4 Protocol Amendments and Dissemination

The 24‐h postoperative corneal assessment (T2) and all other protocol changes introduced during peer review will be submitted to and approved by the Research Ethics Committee and updated in the trial registry before the first participant is enrolled. No participant will be enrolled until both the ethics approval and registry update are complete. The Research Ethics Committee approved Protocol Version 4 on 29 April 2025. Any subsequent substantial modifications will likewise be submitted for approval, communicated to the research team and updated in the trial registry before implementation. Study findings will be reported regardless of their direction or statistical significance and disseminated through a peer‐reviewed open‐access journal and scientific and clinical forums. An accessible summary may also be shared with participants and the public. Neutral or negative findings will be interpreted considering confidence intervals, adherence, event rates, outcome validity and generalisability, rather than as evidence of equivalence.

## 5 Anticipated Outcomes and Implications for Nursing

This pilot and feasibility trial is expected to provide information on recruitment, retention, intervention adherence, completeness of the immediate and 24‐h postoperative corneal assessments, device performance, safety and preliminary corneal‐injury event rates in the perioperative setting. The prespecified progression criteria will be used to determine whether a future definitive trial should proceed unchanged, proceed after targeted protocol modification or be reconsidered. These findings will also be used to refine procedures and inform the design, effect assumptions and sample‐size calculation of a future definitive randomised trial. The study may support the development of evidence‐based perioperative nursing protocols for eye protection.

## 6 Strengths and Limitations

The main strengths of this pilot trial include randomised allocation, identical baseline ocular lubrication in both groups, blinded outcome assessment, objective evaluation of corneal injury using fluorescein staining and slit‐lamp examination at baseline, immediately after surgery and 24 h postoperatively, and prospective collection of feasibility measures including recruitment, retention, adherence, device displacement/reapplication and adverse events. The study will generate perioperative feasibility and event‐rate estimates specific to the eye protection chamber to inform a definitive trial.

Important limitations include the single‐centre design, the inability to blind nurses delivering the visibly different interventions and the limited sample size, which is not intended to establish or exclude superiority. The addition of a 24‐h postoperative assessment improves outcome ascertainment by allowing detection of epithelial injuries that may not be apparent during the immediate postoperative examination; however, the study remains a pilot and feasibility trial and its clinical effect estimates will be interpreted as preliminary.

## Author Contributions

**Thalita Gomes do Carmo:** conceptualization, methodology, software, data curation, investigation, validation, formal analysis, supervision, funding acquisition, visualization, project administration, resources, writing – original draft, writing – review and editing. **Roberto de Souza:** data curation, investigation, writing – original draft, writing – review and editing. **Anna Beatriz Justen dos Santos:** data curation, investigation, writing – original draft, writing – review and editing. **Luana Faria Barbosa:** data curation, investigation, writing – original draft, writing – review and editing. **Giovanna Oliveira de Marteloti:** data curation, investigation, writing – original draft, writing – review and editing. **Maria Eduarda Maciel Palmares:** data curation, investigation, writing – original draft, writing – review and editing. **Eduardo Melani Rocha:** data curation, investigation, writing – original draft, writing – review and editing. **Patricia Rezende do Prado:** conceptualization, methodology, software, data curation, investigation, validation, formal analysis, supervision, funding acquisition, visualization, project administration, resources, writing – original draft, writing – review and editing.

## Funding

This study received financial support from the National Council for Scientific and Technological Development (Conselho Nacional de Desenvolvimento Científico e Tecnológico [CNPq]), Brazil.

## Disclosure

ReBEC is a publicly accessible online platform for the registration of experimental studies involving human participants conducted in Brazil, developed in partnership with the Brazilian Ministry of Health (DECIT/MS), the Pan American Health Organisation (PAHO), and the Oswaldo Cruz Foundation (FIOCRUZ). The ReBEC Executive Committee is composed of the institutions and the Brazilian Health Regulatory Agency (Agência Nacional de Vigilância Sanitária [ANVISA]).

## Ethics Statement

The study was submitted to the Research Ethics Committee of the Faculty of Medicine at Fluminense Federal University and approved on April 29, 2025 (approval number 7.535.554).

## Conflicts of Interest

The authors declare no conflicts of interest.

## Data Availability

The statistical analysis procedures are described in the present protocol. No separate statistical analysis plan will be publicly shared. Individual participant‐level data, including de‐identified data, the study data dictionary and analytic files or statistical syntax, will not be publicly released under the current data‐management and confidentiality plan. Any future controlled‐access sharing would require consistency with participant consent and prior institutional and ethical approval.
